# Who Should Have Charge of the Clinic?

**Published:** 1912-03-02

**Authors:** 


### THE SCHOOL CLINIC.

### Who Should Have Charge of the Clinic?

As the medical treatment of school children is becoming an urgent question of the day there has arisen a cry among many general practitioners that the educational authorities are taking upon themselves the organisation of work which has formerly been in the hands of the general practitioner, that he is capable of doing the work, and therefore that it of right belongs to him and should be put in his hands.

There is another side to the question.

The medical treatment of school children only takes work away from the general practitioner in so far that it improves the health of the nation and so diminishes the number of minor maladies that he has to treat. Again, though there may be found in many districts men capable of doing this work, the general practitioner is not the best person to whom it may be confided. An education authority must consider only what is best for the children under its care. It is a great characteristic of our profession that it is continually trying to better the general health of the nation so as to give itself less work to do, and the criterion of its efficiency is not the amount of treatment performed as a whole, but the amount of unnecessary illness that it prevents, and therefore the number of cases which it keeps away from its own doors. And we do not think that any member of our profession will cavil at the treatment of school children merely because he has fewer common colds or cases of dyspepsia to treat. No one will have the temerity to claim that he has any right to do work which another man may be proved to do better.

### Where the G.P. is Wanting.

The whole question depends upon whether the general practitioner is the best man to do this work or not. The clinics which are rising are devoted each to some special type of disease, and therefore some degree of special education in the work is necessary. The other essentials in a school clinic doctor should be absolute regularity of attendance and a scientific interest in the after-results of his treatment. In these very points the general practitioner is found wanting. The very nature of his work prevents that absolute regularity of attendance which is so essential to the success of all organised methods of treatment. If a man is due at a clinic at 9 A.M. or 1.30 P.M. he cannot leave a midwifery case just as the baby is being born in order to go there. Or if he have an urgent call just as he is starting off he cannot well help answering it. Again, if he have an anxious case he must leave the clinic early if he be summoned to it, because he knows his presence is needed.

The number of general practitioners who have had special experience of any disease is not great; there may be one here and there who has done the post of clinical assistant at, say, some throat hospital, but that period was probably some time ago, and it must needs take a year or more at the work before his special qualifications have returned to him. We do not mean to infer that the general practitioner is not stimulated by the scientific side of his work, but we do say that he has seldom time to develop it, and this interest in the children individually and collectively can only be kept up by a man who has plenty of time to do the work, and who knows he need not be hurried when he has once started his clinic.

### The Post for Young Men.

But if the general practitioner is not the man to do this work, who is to do it? It may be taken for granted that no specialist with established reputation will be found to undertake the work at the price that the education authorities can afford. There is, however, a class of men peculiarly adapted to these positions. We refer to the young men who have lately qualified, who have done their house appointments, and who are spending those years, two to five in number, which most men pass through before they finally settle down in some particular branch of practice. They are most of them filling posts as clinical assistants at one of the special hospitals or hold some junior teaching post at a hospital with medical school attached; others, again, are merely reading for a higher examination.

One and all, however, have some time upon their hands and could easily afford two or more afternoons a week without fear of being called away. Again, they find it as great a struggle to live as does the hardest working practitioner, and any payment that would attract the latter would be of value to them, and if any question of claim be put in they can say that they do much gratuitous work at the hospitals, and therefore have as much claim upon the State as the general practitioner who says his work is being taken away from him. But above all they have recent specialised training as dressers or as clinical assistants in some recognised special department and are fired with a desire for research and to follow through to the end the cases they have seen in order to test the truth of what they have heard from their teachers.

### The "Embryo Consultant."

It is the fashion in some quarters to speak disparagingly of these men as "embryo consultants," but as many develop into general practitioners as ever pass into the ranks of the consultants, and it is better to call them men engaged in post-graduate study. This study is the most valuable which a man ever goes through, in which he is teaching himself and sifting the things he has heard and read in his student days. And while we believe that they are the men and women eminently fitted for the posts, we also believe that the State would be practising an ultimate economy by increasing the number who can afford to spend their time at this work and so increase the efficiency of the profession as a whole.

